# Improving the estimation of the tuberculosis burden in India

**DOI:** 10.2471/BLT.13.129775

**Published:** 2014-08-20

**Authors:** Krycia Cowling, Rakhi Dandona, Lalit Dandona

**Affiliations:** a Public Health Foundation of India, Plot 47, Sector 44, Gurgaon, 122002, National Capital Region, India.

## Abstract

Although India is considered to be the country with the greatest tuberculosis burden, estimates of the disease’s incidence, prevalence and mortality in India rely on sparse data with substantial uncertainty. The relevant available data are less reliable than those from countries that have recently improved systems for case reporting or recently invested in national surveys of tuberculosis prevalence. We explored ways to improve the estimation of the tuberculosis burden in India. We focused on case notification data – among the most reliable data available – and ways to investigate the associated level of underreporting, as well as the need for a national tuberculosis prevalence survey. We discuss several recent developments – i.e. changes in national policies relating to tuberculosis, World Health Organization guidelines for the investigation of the disease, and a rapid diagnostic test – that should improve data collection for the estimation of the tuberculosis burden in India and elsewhere. We recommend the implementation of an inventory study in India to assess the underreporting of tuberculosis cases, as well as a national survey of tuberculosis prevalence. A national assessment of drug resistance in Indian strains of *Mycobacterium tuberculosis* should also be considered. The results of such studies will be vital for the accurate monitoring of tuberculosis control efforts in India and globally.

## Résumé

Bien que l'Inde soit considérée comme le pays le plus touché par la tuberculose, les estimations de l'incidence, la prévalence et la mortalité relative à cette maladie en Inde reposent sur des données insuffisantes marquées d'une forte incertitude. Les données disponibles pertinentes sont moins fiables que celles obtenues dans les pays qui ont récemment amélioré leur système de signalement de cas ou qui ont récemment investi dans des enquêtes nationales de prévalence de la tuberculose. Nous avons exploré les moyens d'améliorer l'estimation de la charge de morbidité de la tuberculose en Inde. Nous nous sommes concentrés sur les données de signalisation des cas (parmi les données les plus fiables disponibles) et sur les moyens d'étudier les niveaux associés de sous-déclaration, ainsi que le besoin d'une enquête nationale de prévalence de la tuberculose. Nous discutons de plusieurs développements récents (c.-à-d. les changements dans les politiques nationales relatives à la tuberculose, les recommandations de l'Organisation mondiale de la Santé pour la recherche sur la maladie et un test diagnostique rapide) qui devraient améliorer la collecte des données pour estimer la charge de morbidité de la tuberculose en Inde et ailleurs dans le monde. Il serait utile de mettre en œuvre une étude d'inventaire pour évaluer la sous-déclaration des cas de tuberculose, une étude de la prévalence de la tuberculose et une évaluation de la résistance aux médicaments des souches indiennes de *Mycobacterium tuberculosis.* Les résultats de ces études seront d'une importance vitale pour le suivi précis des efforts de contrôle de la tuberculose en Inde et dans le monde.

## Resumen

Aunque la India se considera el país con la mayor carga de tuberculosis, las estimaciones de la incidencia, la prevalencia y la mortalidad de la enfermedad en el país se basan en datos escasos con falta de fiabilidad considerable. Los datos disponibles pertinentes son menos fiables que los datos de los países que han mejorado recientemente los sistemas de notificación de casos o que han invertido últimamente en encuestas nacionales de prevalencia de la tuberculosis. Se estudiaron los modos de mejorar la estimación de la carga de tuberculosis en la India, con un enfoque en los datos de la notificación de casos, que se encuentran entre los datos más fiables disponibles, y las formas de analizar la correspondiente falta de notificación de casos, así como en la necesidad de una encuesta nacional de prevalencia de la tuberculosis. Se examinaron varios acontecimientos recientes, es decir, los cambios en las políticas nacionales en materia de tuberculosis y las directrices de la Organización Mundial de la Salud para la investigación de la enfermedad, así como una prueba de diagnóstico rápido, que se espera que mejoren la recogida de datos para la estimación de la carga de la tuberculosis en la India y otros lugares. Sería conveniente realizar un estudio de inventario para evaluar la falta de notificación de casos de tuberculosis, una encuesta sobre la prevalencia de la tuberculosis y una evaluación de la resistencia a fármacos en cepas de *Mycobacterium tuberculosis* de la India*.* Los resultados de estos estudios serán vitales para el seguimiento preciso de los esfuerzos de control de la tuberculosis, tanto en la India como en el resto del mundo.

## ملخص

رغم أن الهند تعتبر البلد الذي يرزح تحت عبء السل الأشد وطأة، إلا أن تقديرات معدل الإصابة بالمرض وانتشاره ومعدل وفياته في الهند تعتمد على بيانات شحيحة غير مؤكدة بشكل كبير. وتعتبر البيانات المتاحة ذات الصلة أقل موثوقية من تلك المستمدة من البلدان التي قامت في الآونة الأخيرة بتحسين نظم الإبلاغ عن الحالات أو قامت بالاستثمار في الدراسات الاستقصائية الوطنية المعنية بانتشار السل. وقمنا باستكشاف طرق تحسين تقدير عبء السل في الهند. وركزنا على بيانات الإبلاغ عن الحالات - بين أكثر البيانات المتاحة موثوقية - وطرق تحري مستوى نقص الإبلاغ ذي الصلة، بالإضافة إلى الحاجة إلى دراسة استقصائية وطنية عن انتشار السل. ونناقش العديد من التطورات الحديثة - أي التغيرات في السياسات الوطنية ذات الصلة بالسل والمبادئ التوجيهية لمنظمة الصحة العالمية من أجل تحري المرض وإجراء اختبار تشخيصي سريع - التي ينبغي أن تحسن جمع البيانات بغية تقدير عبء السل في الهند وفي غيرها من البلدان. وسيكون من المفيد تنفيذ دراسة حصرية لتقييم نقص الإبلاغ عن حالات السل، ودراسة استقصائية عن انتشار السل وتقييم مقاومة الأدوية في سلالات البكتريا المتفطرة السليّة في الهند. وستكون لنتائج هذه الدراسات أهمية بالغة من أجل الرصد الدقيق لجهود مكافحة السل، في الهند وعلى الصعيد العالمي.

## 摘要

尽管印度被认为是结核病负担最重的国家,但是印度疾病的发病率、患病率和死亡率估计依赖大量不确定的稀疏数据。印度相关数据的可靠性低于新近改善了病例报告系统或者最近对结核病患病率国家调查进行投入的国家。我们探讨印度改善结核病负担估算的方法。我们把重点放在(最可靠的可用数据中)病例通报数据和相关漏报水平调查方法以及是否需要全国性结核病患病率调查上。我们讨论可以改善印度和其他地方结核病负担估计的数据收集方面的最近发展,即国家肺结核政策方面的变化、世界卫生组织疾病调查指导方针和一种快速诊断测试。实施评估结核病病例漏报的详细目录研究、结核病患病率的调查以及印度分枝杆菌结核病菌株耐药性评估将非常有用。这些研究的结果将对印度和全球准确监测结核病的控制工作至关重要。

## Резюме

Хотя Индия считается страной с наиболее высоким уровнем заболеваемости туберкулезом, однако оценки заболеваемости, распространения и уровня смертности в Индии основаны на недостаточном количестве данных, а в доступных данных присутствует значительноеколичество неточностей. Доступные данные менее достоверны, чем в других странах, где была усовершенствована система сообщения о случаях заболевания и где осуществлялись инвестиции в национальные исследования распространения туберкулеза. В данной работе проведено исследование способов усовершенствования оценки уровня заболеваемости туберкулезом в Индии. Особое внимание уделялось данным о зафиксированных случаях заболевания – наиболее надежных среди доступных данных – и способам исследования уровня занижения показателей, а также необходимости национального исследования заболеваемости туберкулезом. Были рассмотрены несколько недавних разработок, например, изменения национальной политики в отношении туберкулеза, применение указаний Всемирной организации здравоохранения по исследованию заболевания и быстрые методы диагностики, которые помогут улучшить сбор данных для осуществления оценки уровня заболеваемости туберкулезом в Индии и других странах. Необходимо провести исследование для учета данных, которое поможет определить уровень занижения показателей по зафиксированным случаям заболевания туберкулезом, исследование распространенности туберкулеза и оценку устойчивости к лекарственным средствам штаммов *Mycobacterium tuberculosis,* распространенных в Индии. Результаты таких исследований будут иметь жизненно важное значение для борьбы с туберкулезом в Индии и во всем мире.

## Introduction

India accounts for an estimated 2.2 million of the 8.6 million new cases of tuberculosis that occur each year globally and harbours more than twice as many cases as any other country.^1^ A national programme for the control of tuberculosis achieved nationwide coverage in 2006 but this programme has limitations in terms of disease surveillance.^2^ All attempts to estimate the burden of tuberculosis in India are based on indirect methods characterized by substantial uncertainty and a lack of subnational detail. For a country of over 1.2 billion people, including, probably, more than 500 million individuals latently infected with *Mycobacterium tuberculosis*, weaknesses in the available estimates of the tuberculosis burden are disappointing and limit effective policy-making.^3^ China – the country with the second-highest number of tuberculosis cases – has drastically improved its tuberculosis estimates since implementing a web-based system of mandatory case reporting, in 2005.^4^ More accurate estimates of the tuberculosis burden in India are needed to guide national policy-making, to improve the assessment of control efforts and to understand global trends in the incidence of tuberculosis. Here, we review the data currently used to estimate the tuberculosis burden in India and their limitations, and discuss options for the collection of new data that could yield improved estimates.

## Estimating the tuberculosis burden

### Possible sources

Ideally, in any country, tuberculosis surveillance is based on a comprehensive monitoring system to which all new cases are reported and a vital registration system that collects accurate data on the causes of all deaths. Such case monitoring and vital registration systems allow evaluation of the incidence of new infections and the levels of tuberculosis-related mortality, respectively. In countries lacking such systems, estimates of the tuberculosis burden are typically based on national tuberculosis prevalence surveys and national mortality surveys that assess causes of death. Although many countries have either undertaken prevalence surveys since 2002 or are planning to undertake such surveys by 2017,^1^ India is not one of them (see Appendix A, available at http://www.phfi.org/images/Publications/journals/2014_who_bulletin%20_tb_estimation_india_web_appendix_a.pdf). India has not implemented a national survey of tuberculosis prevalence since 1955.^5^ Tuberculin surveys – which allow the prevalence of latent *M. tuberculosis* infection to be estimated – are no longer recommended by the World Health Organization (WHO) for estimating the prevalence of active tuberculosis.^6^^,^^7^

### Data availability in India

India’s current efforts and investments in generating reliable data for estimating its tuberculosis burden are inadequate, especially when compared with the corresponding efforts in countries with similar levels of wealth and tuberculosis endemicity. India does not yet have comprehensive systems for the reporting of tuberculosis cases or vital registration and it has only conducted scattered subnational surveys on the prevalence, incidence and mortality of tuberculosis. Although the Revised National Tuberculosis Control Programme is working to improve the system for the notification of tuberculosis cases in India, it will be many years before every relevant provider is participating. The improvement of India’s vital registration system is an important larger goal that will also take a long time. In addition, there is no commitment to conduct a national survey on the prevalence of tuberculosis in India, even though it is over 59 years since such a survey was last conducted.^5^

Currently, estimates of the tuberculosis burden in India are predominately based on the numbers of cases that are notified and expert opinion on the corresponding level of underreporting. Such estimates could be made more accurate and less subjective if expert opinions could be replaced with empirical estimates of the level of underreporting in the case notification system.

**Fig. 1:**
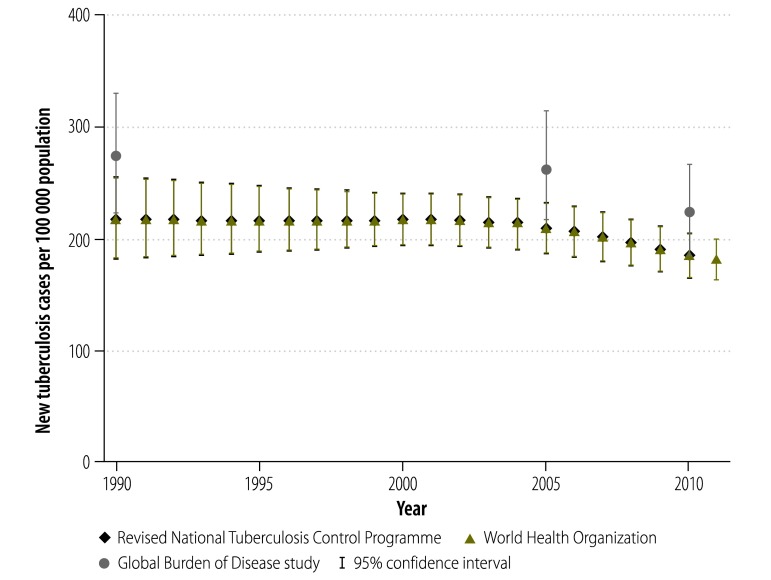
Estimates of the mean incidence of tuberculosis, India, 1990–2011

Note: The estimates from the Global Burden of Disease Study exclude cases that test positive for the human immunodeficiency virus.

**Fig. 2:**
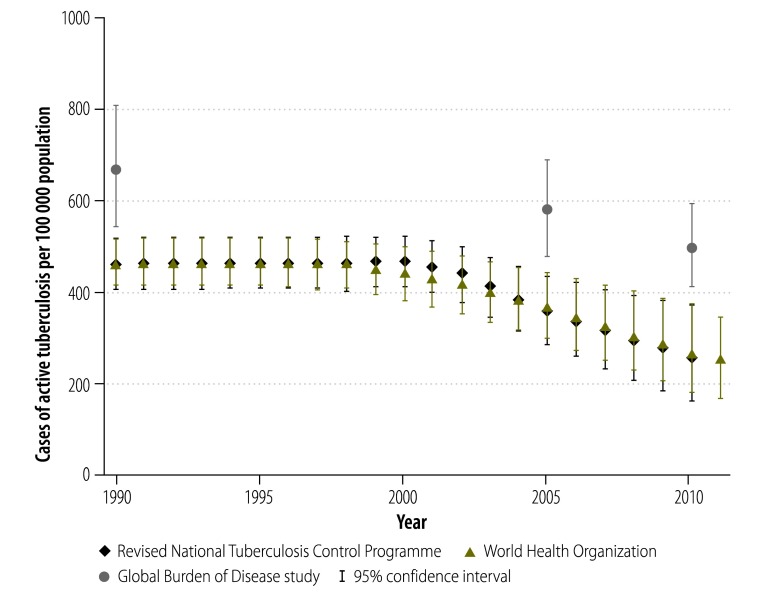
Estimates of the mean prevalence of tuberculosis, India, 1990–2011

Note: The estimates from the Global Burden of Disease Study exclude cases that test positive for the human immunodeficiency virus.

**Fig. 3:**
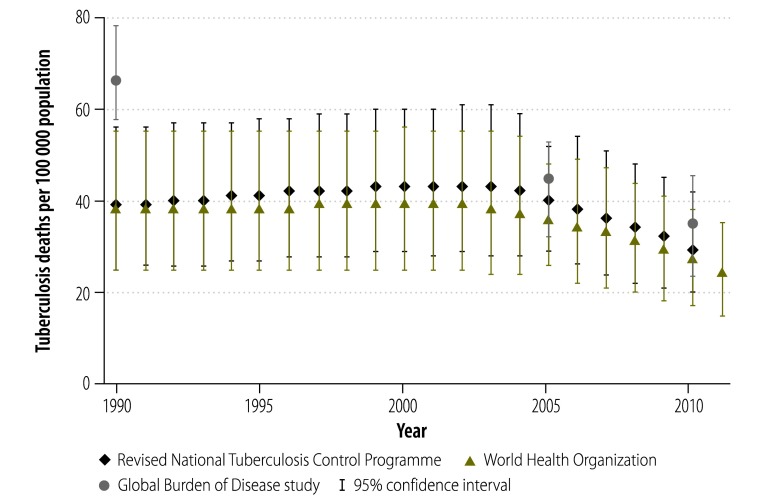
Estimates of the mean level of tuberculosis-attributable mortality, India, 1990–2011

Note: Deaths caused by co-infection with the human immunodeficiency virus and tuberculosis have been excluded.

The most widely-used estimates of the national burden of tuberculosis in India are produced by the Revised National Tuberculosis Control Programme, WHO and the Global Burden of Disease Study.^2^^,^^8^^,^^9^ The uncertainty in these estimates is illustrated by comparing the values for tuberculosis incidence (Fig. 1), prevalence (Fig. 2) and mortality (Fig. 3) in India from these three sources for the period 1990–2011. The Revised National Tuberculosis Control Programme and WHO produce their estimates of incidence by dividing the number of case notifications by 1 minus the estimated proportion of all cases that are not reported.^8^ The level of underreporting has been estimated from expert opinions and from the results of two subnational studies – which indicated that only about 60% of tuberculosis cases in the study areas were notified.^10^^,^^11^ The Global Burden of Disease Study used substantially different methods and several covariates to try to strengthen estimates based on sparse data.^12^ Greater details for each methodology – and a discussion of the difficulties of measuring the success of the Revised National Tuberculosis Control Programme using rates of case detection – are provided in Appendix A.

## Underreporting

### The private sector

Although case notification data are routinely collected in all of India’s districts, the corresponding levels of underreporting – and the geographical variation in those levels – are unknown. The Revised National Tuberculosis Control Programme has nationwide coverage of the public providers of health care but only limited engagement with the private sector – where at least 50% of tuberculosis patients are estimated to seek treatment.^13^^–^^16^ In a 2011 survey in 30 low-performing districts representative of those receiving support from the Global Fund to Fight AIDS, Tuberculosis and Malaria, about half of the members of study households needing tuberculosis treatment went to private providers.^10^ Smaller studies assessing the proportion of cases treated in the private sector in India are discussed in Appendix A. In 2012, India declared tuberculosis a notifiable disease – i.e. it made the reporting of tuberculosis mandatory. However, there remains much scope to improve notifications from private providers, partly because of the generally poor regulation of the private sector.^17^^,^^18^ Public–private mix initiatives are interventions intended to educate and engage private providers in diagnosing, reporting and treating tuberculosis in accordance with national guidelines.^19^ Such initiatives have been implemented in India but only currently cover 14 major cities and about 50 million people. This represents the lowest coverage of the 20 countries in which coverage with such initiatives has been reported.^20^ In areas of India covered by these initiatives, 45% of all new smear-positive cases of tuberculosis are reported by private providers.

### Framework to assess underreporting

**Fig. 4:**
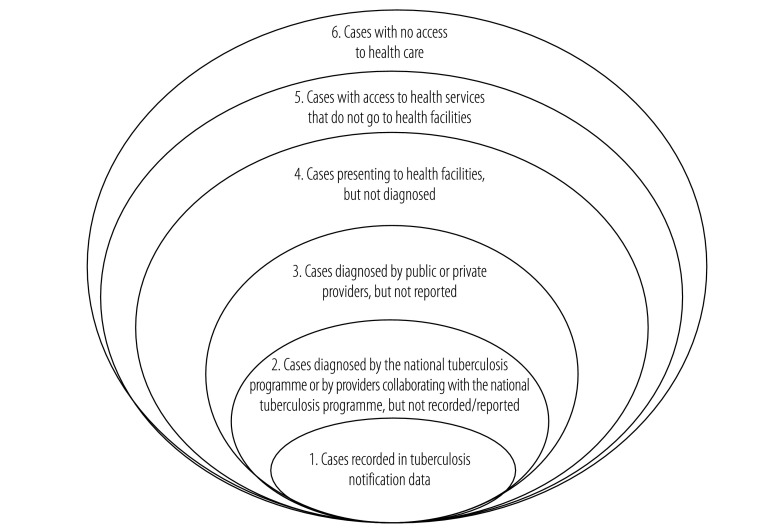
World Health Organization “onion” model for assessing the fraction of tuberculosis cases missed by routine notification data

Source: World Health Organization.^21^

In addition to cases not reported by private providers, other factors also contribute to underreporting of tuberculosis cases. In 2002, WHO developed the “onion” model as a framework for assessing the percentage of tuberculosis cases that go unreported (Fig. 4).^21^ The model’s six rings range from cases in people with no access to the health system – representing the sixth and outermost layer of the “onion” – to those diagnosed and reported by providers affiliated to a national tuberculosis programme – representing the first and innermost layer and the only cases captured in case notification data. A variety of data sources can be used to estimate the proportion of cases in each ring. Proportions differ by country because of variations in national health systems.

### Data to assess underreporting

#### Cases outside the health system

The two outermost rings of the “onion” model correspond to the proportion of the population without access to the health system and the proportion that does not utilize health services even though they have access. Together, these two rings are populated by people who do not seek treatment from the health system. In India, in the 2007–2009 District Level Household Survey, 2584 (0.34%; 95% confidence interval, CI: 0.33–0.36%) of the 717 691 study households reported that they did not seek any form of medical treatment when their members were sick. The subnational variation in the proportion of cases not accessing the health system can be evaluated from data collected in District Level Household Surveys.

#### Underdiagnosis

Different types of proxy data currently represent the best available options for estimating the proportion of cases in the fourth ring of the onion model – i.e. cases that present to providers but are not correctly diagnosed. Although all undiagnosed cases are combined in the onion model, in India it is useful to distinguish between the undiagnosed cases who present to providers affiliated to the Revised National Tuberculosis Control Programme and those who present to other providers. There is more relevant information available on the programme-affiliated providers and these providers should have relatively lower rates of underdiagnosis because they are expected to adhere to certain diagnostic algorithms.^22^^,^^23^ In general, providers who are not affiliated to the programme rely heavily on radiology for investigating a potential case of tuberculosis, even though radiology has low specificity and is more appropriate as a screening tool than as a diagnostic test.^24^ The programme’s diagnostic algorithms prescribe the use of sputum-smear microscopy, which has a sensitivity of about 64% and a specificity of approximately 98%.^25^ Diagnostic performance can be enhanced by repeating such microscopy for all symptomatic patients who have been initially found smear-negative – a procedure that is also recommended by the programme.^25^ The level of underdiagnosis may be reasonably approximated from programme data that are released quarterly for each district. These data include the number of suspects examined per smear-positive case diagnosed as well as scores – based on performance monitoring – for the district’s case-finding efforts. Although WHO has suggested that underdiagnosis might be assessed by evaluating laboratory capacities or the knowledge and practices of health staff, there is sparse literature to support the accuracy or feasibility of these methods, which need further development.^26^

#### Inventory studies

Individuals in the second and third rings of the onion model – i.e. cases that are correctly diagnosed but not reported – can be investigated through inventory studies.^20^ The need for such studies in India was highlighted in the 2011 WHO global tuberculosis report.^20^ WHO recently published a guide for countries undertaking inventory studies,^27^ which can be used for one of three objectives: to quantify the level of underreporting of diagnosed cases, to estimate tuberculosis incidence by using capture–recapture methods, or to demonstrate that the underreporting of diagnosed cases is negligible. The WHO guide describes four possible designs for an inventory study, with the choice depending on the chosen objective or objectives and the available data (Table 1): a survey of all providers in randomly sampled areas; a survey of all providers in large self-contained areas – with at least two additional case databases; a retrospective analysis with no new data collection; and a survey of a sample of all providers selected using lot-quality assurance sampling. Each of these types of inventory study can be greatly facilitated by an existing national database of diagnosed cases with unique identifiers and standard case definitions. India is currently scaling up its web-based system of case reporting and planning the use of unique identification numbers for all diagnosed cases.^28^ However, the limited engagement of the Revised National Tuberculosis Control Programme with the private sector may limit the usefulness of these new initiatives. If individuals in multiple databases are assigned more than one unique identification number, cases may be linked by probabilistic matching. Though error-prone, this has been used successfully in related studies.^29^

**Table 1:** Possible study designs for estimating the tuberculosis burden in India

Study design	Possible objectives	Existing data used	New data collection required	Current feasibility	Application	
					Advantages	Disadvantages
**Inventory study**						
Retrospective analysis^a^	Quantification of underreporting of diagnosed cases. Estimation of tuberculosis incidence. Demonstration of negligible underreporting	National tuberculosis surveillance database plus one or two national case-based databases – the exact number depending on objectives	None	Not feasible because multiple national case-based databases not available in India		
Survey of sample of all providers, selected using lot-quality assurance sampling^a^	Demonstration of negligible underreporting	National tuberculosis surveillance database	Provider survey of random sample of all tuberculosis providers, selected using lot-quality assurance sampling	Not appropriate because underreporting known to be substantial in India		
Survey of all providers in large areas suitable for capture–recapture analysis^a^	Quantification of underreporting of diagnosed cases. Estimation of tuberculosis incidence	National tuberculosis surveillance database plus two other case-based databases for each geographical area selected	Provider survey of all tuberculosis providers in random sample of large, self-contained geographical areas	Needs to be assessed	Generates comprehensive, direct estimate of underreporting at all levels	Assumptions regarding migration and probability of inclusion in each database. Error-prone because of reliance on probabilistic matching across multiple databases
Survey of all providers in sampled areas^a^	Quantification of underreporting of diagnosed cases. Demonstration of negligible underreporting	National tuberculosis surveillance database	Provider survey of all tuberculosis providers in random sample of geographical areas	Feasible for quantifying underreporting of diagnosed cases	Of the feasible studies, relatively inexpensive because fewer data need to be collected	Proportion of cases with no health system utilization estimated from self-reported household survey data. Level of underdiagnosis estimated from other new data collection or existing data with limitations
Survey of all providers in sampled areas with assessment of underdiagnosis	Quantification of underreporting and underdiagnosis by RNTCP and non-RNTCP providers	National tuberculosis surveillance database	Provider survey of all tuberculosis providers in random sample of geographical areas, including assessment of underdiagnosis	Feasible	Generates direct estimates of the greatest number of the parameters contributing to underreporting	Proportion of cases with no health system utilization estimated from self-reported household survey data. More expensive than assessment of only underreporting of diagnosed cases in sampled areas because of additional data collection
**National prevalence survey**	Estimation of national prevalence of active tuberculosis in adults. Assessment of the proportion of tuberculosis cases which are drug-resistant	None	For nationally representative sample of adults aged ≥ 15 years: either Xpert MTB/RIF assay or X-ray screening plus two sputum samples if symptomatic or X-ray abnormal	Feasible	Generates direct estimate of national tuberculosis prevalence, with potential to assess extent of drug resistance	In comparison with other study designs, longer period of data collection and more expensive

RNTCP: Revised National Tuberculosis Control Programme.

^a^ Described in detail in the WHO guide for conducting inventory studies.^27^

For India, given the current status of the national case reporting system, a survey of all providers in randomly sampled areas is the most applicable of the feasible types of inventory study. The capture–recapture analysis using a survey of all providers in large, self-contained areas has fairly stringent data requirements, including at least three independent registries across which record linkage is possible, the right degree of overlap between these registries – ideally 15–30% – and a population with little to no migration and an equal probability of a case being recorded in each registry. These requirements cannot be met at the national level in India at this time. India’s National Tuberculosis Institute is conducting a capture–recapture study in Tumkur district, Karnataka state, and the methods used in that study should be assessed for their possible use at the national level. The methods used in recent capture–recapture studies conducted in other low- and middle-income countries (Appendix A) may also provide indications of the possibility of such studies in India.

Table 2 summarizes the data sources that might be used in India to estimate each component of underreporting. In addition to quantifying the underreporting of diagnosed cases by all providers, an inventory study would also generate empirical estimates of the proportions of tuberculosis cases that are treated by providers affiliated and not affiliated to the Revised National Tuberculosis Control Programme. Given the limitations of the existing data sources for estimating underdiagnosis in India, the value of any inventory study is likely to be enhanced by the collection of new data on underdiagnosis by all providers (Table 1 and Table 2). The providers followed to assess underreporting could also be evaluated for underdiagnosis, either by tracking the proportion of patients presenting with symptoms indicative of tuberculosis for whom providers order diagnostic tests^30^ or using medical vignettes.^31^ Although such studies are reliant on the cooperation of the relevant providers, such assistance has been obtained in earlier studies in India.^32^^–^^34^

**Table 2:** Data needed, in each of the feasible types of inventory study, to estimate underreporting of tuberculosis cases in India

Characteristics of tuberculosis case	Data sources		
	Survey of all providers in sampled areas	Survey of all providers in large areas suitable for capture–recapture analysis	Survey of all providers with assessment of underdiagnosis
**Not reported to the case notification system**			
No access to health system	Household surveys	Provider survey with capture–recapture analysis	Household surveys
No utilization of health system	Household surveys		Household surveys
Using non-RNTCP providers:			Provider survey
Not diagnosed	Assessment of laboratory capacity or knowledge and practices of health staff		
Diagnosed but not reported	Provider survey		
Using RNTCP providers:			
Not diagnosed	RNTCP case-finding efforts score or the number of patients examined per case		
Diagnosed but not reported	Provider survey		
**Diagnosed and reported to the case notification system**	Case notifications	Case notifications	Case notifications

RNTCP: Revised National Tuberculosis Control Programme.

## National prevalence surveys

At this time there appears to be no widespread governmental support for a national survey of tuberculosis prevalence in India. In an analogous situation less than a decade ago, a national human immunodeficiency virus (HIV) prevalence survey was not recommended for India but, when undertaken, led to a huge adjustment in the estimated HIV burden in India.^35^

### Embedded or stand-alone surveys?

A national survey of tuberculosis prevalence could be embedded within one of the nationwide household surveys periodically conducted in India, which would probably be more cost–effective than a stand-alone prevalence survey. Although existing household surveys cover a small proportion of Indian households, India’s vast population ensures that such surveys provide adequate samples for assessing tuberculosis prevalence, even at subnational levels. For example, the 2005–2006 National Family Health Survey sampled almost 110 000 households.^36^ Furthermore, the nationwide household surveys use cluster designs, which are recommended for prevalence surveys.^37^ Recent National Family Health Surveys and District Level Household Surveys have collected data on self-reported tuberculosis but these are considered too inaccurate for estimating tuberculosis burdens (Appendix A).

One advantage of a stand-alone survey is that it can be scheduled for the near future. Plans for the next National Family Health Survey, in 2014–2015, are too advanced for the inclusion of a tuberculosis prevalence survey to be considered. The subsequent National Family Health Survey will probably not be implemented until at least 2019. A stand-alone tuberculosis prevalence survey could also allow tuberculosis-related risk factors and issues of health-care access to be explored more completely than might be feasible in a general national health survey. India’s size and diversity pose challenges for all national surveys; however, successful periodic household surveys on many health topics and a successful nationwide assessment of HIV prevalence indicate the general feasibility of a national survey of tuberculosis prevalence.

### Drug resistance

The planners of any future nationwide prevalence survey should consider the use of the new Xpert MTB/RIF assay (Cepheid, Sunnyvale, United States of America), which detects both pulmonary and extrapulmonary tuberculosis, provides results in less than 2 hours and simultaneously tests for drug resistance.^38^ In field tests among smear-negative but culture-positive patients, the assay demonstrated a sensitivity of 77% and a specificity of 99%.^39^ Although the assay is easy to use with sputum samples and provides rapid results, it is costly and its use may pose logistical challenges in some settings – at least initially. The current form of the assay only detects resistance to rifampicin but data on the distribution among tuberculosis cases in India of resistance to just this drug may still provide useful insights. However, there has been some recent concern about the assay’s accuracy in detecting rifampicin resistance in India.^40^

A better understanding of the prevalence and distribution of drug-resistant tuberculosis is an emerging priority. Escalating prevalences of multidrug-resistant and extensively drug-resistant tuberculosis are among the greatest challenges to tuberculosis control globally – and India has the greatest number of cases of these forms of tuberculosis.^8^ Subnational studies have revealed alarmingly high and increasing prevalences of multidrug-resistant tuberculosis in some areas of India.^41^ Any comprehensive investment in the collection of better data for estimating the tuberculosis burden in India should therefore include some support for the evaluation of the role of drug resistance.

## Recommendations

This discussion outlines several options for the collection of new data to improve estimates of the tuberculosis burden in India. We recommend that both a study of underreporting in the case notification data and a national tuberculosis prevalence survey – possibly including an assessment of drug resistance – be implemented soon.

An inventory study to assess underreporting of tuberculosis cases – based on the new WHO manual for such studies^27^ – should be planned. Although substantial data collection would be required, an inventory study could be relatively short. WHO recommends only three months of follow-up for such a study. We believe a survey of all providers in randomly sampled areas, including an assessment of underdiagnosis, would be the best option because it would generate empirical estimates for the greatest number of relevant parameters. Regardless of the study design used, the sampling of a large number of diverse areas is critical to elucidating any subnational variation in India’s tuberculosis burden.

While logistically more demanding than an inventory study, a prevalence survey would be complementary. If both types of study were implemented, our understanding of tuberculosis epidemiology and control efforts in India would improve further. The costs and benefits of embedded and stand-alone prevalence surveys and the value of a simultaneous assessment of drug resistance should be carefully weighed.

Investments in new data are particularly important for understanding the subnational variation in tuberculosis epidemiology within India’s large population. Improved estimates for India would greatly contribute to a better understanding of the global tuberculosis epidemic. Further development of the methods used to assess underreporting –through their application in India – would also benefit other countries wishing to assess the quality of their case reporting systems. Finally, the need for these studies is timely given the recent goals set by the Indian government in the 2012–2017 Revised National Tuberculosis Control Programme Strategic Plan, which require reliable data for planning and evaluation.
